# Determining the effectiveness of cognitive behavioral therapy interventions based on the transdiagnostic approach in the treatment of common mental health problems: Presenting an experience from the Islamic Republic of Iran

**DOI:** 10.1002/brb3.2551

**Published:** 2022-04-04

**Authors:** Katayoun Falahat, Monir Baradarn Eftekhari, Masoumeh Dejman, Ameneh Setareh Forouzan, Zohreh Mahmoodi, Mojgan Padyab, Samira Tavassoli

**Affiliations:** ^1^ Deputy for Research and Technology Ministry of Health and Medical Education Tehran Iran; ^2^ Department of Mental Health Johns Hopkins Bloomberg School of Public Health Baltimore Maryland USA; ^3^ Social Welfare Management Research Center University of Social Welfare and Rehabilitation Sciences Tehran Iran; ^4^ Social Determinants of Health Research Center Alborz University of Medical Sciences Karaj Iran; ^5^ Department of Social Work Umea University Umea Sweden; ^6^ Gallatin School of Individualized Study New York University New York New York USA

**Keywords:** anxiety, cognitive behavioral therapy, depression, Iran, mental health, obsessive compulsive disorder

## Abstract

**Introduction:**

There is growing support to develop transdiagnostic approaches that provide new insights into mental health problems and cut across the existing traditional diagnostic boundaries all over the world. The present study was conducted to test the transdiagnostic cognitive behavioral therapy (TCBT) approach in treating patients with common mental health problems and evaluate its effectiveness compared to the current treatment settings of the healthcare system.

**Methods:**

A randomized controlled trial was conducted in Semnan Province, north of Iran. The study took pace in urban health centers. A sample of 520 Iranian adults, tested as positive on the Kessler Psychological Distress Scale, were enrolled. Participants who received a score above the cut‐off point in any of the three mental health disorders (depression, anxiety, or obsessive compulsive disorder [OCD]) based on the locally validated study instrument were randomly allocated to the study. The intervention group received TCBT during eight sessions provided by trained general health service providers without previous mental health training; the standby control group received Mental Health Services as Usual (MHSU). The post‐test interviews were conducted using the study instrument after the completion of both group treatments.

**Results:**

A total of 459 individuals (87.8% female) ultimately entered the study. The withdrawal rate was 24% (53 participants in the TCBT and 56 in the MHSU). Reduction in depression, anxiety, and OCD symptoms was significant within each group and when comparing TCBT and MHSU (mean difference).

**Conclusion:**

This trial recommends that the transdiagnostic CBT approach can be effective in improving common mental health problems and functions among individuals by trained general healthcare providers in the primary healthcare system. The results can be more useful in decision making when defining the process of providing mental healthcare in the National Primary Healthcare System.

## Graphical abstract

In this study, a randomized controlled trial was conducted to evaluate the effectiveness of trans diagnostic cognitive behavioral therapy on patients with common mental health problems (Depression, Anxiety and OCD). Result indicated statistically significant difference in Depression, Anxiety and OCD symptoms within and between intervention (TCBT) and control (MHSU) groups.

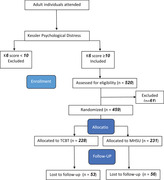

## 1 INTRODUCTION

Mental health problems account for a significant portion of the disease burden worldwide (Patel et al., 2016; Rehm & Shield, 2019). The global prevalence of common mental disorders, including mood disorders, anxiety, and substance abuse, is estimated at 29.2% in adults and its incidence over 12 months was 17.6% (Steel et al., 2014). According to national studies, the prevalence of such disorders is 17.1% to 23.6% in Iran (Moradpour et al., 2019).

These problems are 9th among the 20 leading causes of living with disability and account for 10% of the global disease burden (Chisholm, 2015). Following injuries from external causes, the highest disease burden in Iran is related to mental health problems, which account for 16.4% of the total disease burden (Forouzanfar et al., 2014; Naghavi et al., 2009).

Mental health problems have very adverse effects on the lives of individuals, families, and communities. Nonetheless, only 2% of these patients are treated worldwide, and more than 70% of people in need of mental health services do not have access to such care (Wainberg et al., 2017). This discrepancy becomes more pronounced when effective evidence‐based interventions are carried out in environments with limited resources (Bass et al., 2013).

The extent and importance of these disorders highlight the need to expand access to evidence‐based therapy (EBT) with the aim of implementing this form of therapy by health professionals. Recent developments in psychotherapy interventions in the US and elsewhere have focused on the transdiagnostic approach. This approach uses several EBTs—cognitive‐behavioral therapy (CBT), interpersonal psychotherapy (IPT)—to provide common therapeutic components that can be effective in treating a wide range of problems (Barlow et al., 2016). This approach is expected to go beyond the existing categorized diagnoses and to create a better classification compared to the standard system. This approach is an integrated approach that enables healthcare providers to solve multiple problems with a single treatment (Fusar‐Poli et al., 2019).

The available treatments often require specialized physicians who are trained to treat different problems with different forms of therapy. Moreover, the majority of people who need treatment rarely have only one problem or disorder. Meanwhile, the existing limitations, such as specialists’ lack of time, demonstrate that it might be best for health professionals to use an integrated method, such as the transdiagnostic approach (Barlow et al., 2016; Fusar‐Poli et al., 2019).

One of the transdiagnostic psychotherapy interventions for adults with mood disorder or anxiety problems, especially in low‐income countries, is the Common Elements Treatment Approach (CETA). This approach does not mean a new intervention, but it is a new approach to educate healthcare providers that focuses on the common elements of EBTs and their decision making (Murray et al., 2014). This interventional approach has been adopted in some developing countries such as Thailand and Iraq, and the results indicate the effectiveness of transdiagnostic interventions in the treatment of common mental health problems in the studied communities (Bolton et al., 2014; Murray et al., 2014; Weiss et al., 2015).

Since 1986 in Iran, mental health services have become integrated into the country's primary healthcare (PHC) system (Sharifi, 2009). This process, which begins with the initial screening of mental health problems by healthcare providers, is followed by a pyramid referral to more specialized services if needed. Obviously, despite its benefits, referral to more specialized services is faced with serious problems in terms of the provision of medical services in some cases due to the longer time spent, multiplicity of referrals (Saberi Zafarghandi, 2011), and the stigma of referring to a psychologist or psychiatrist (Taghva et al., 2017). The present study was conducted to test a transdiagnostic cognitive behavioral therapy (TCBT) developed for presentations of common mental health problems among general population and facilitate the provision of mental health services in a shorter time, at a lower cost and with less stigma by employing and training healthcare providers and to evaluate its effectiveness compared to the current treatments.

## 2 MATERIALS AND METHODS

### 2.1 Study site

This study took place in Semnan Province, situated in the north of Iran. The population in this province was estimated at just over 702,000, living in 37,000 households, based on the results of the 2016 national census (Statistical Center of Iran, Planning and Budget Organization, 2018). According to a National Mental Health Survey in 2017, the prevalence of suspected psychiatric disorder cases was 14.5% (15.8% female and 13.1% male). Also, the prevalence rates of depression, anxiety, and somatization were 7.2%, 23.8%, and 20% in this region (Noorbala et al., 2017). This province was selected for this study due to its low rate of immigration, proximity to the capital, and good accessibility for the research team as well as the proper cooperation of the province's PHC system, especially its mental health service providers. With the integration of mental health services into the national PHC system, Semnan Province now has 35 urban health centers (UHCs), 30 rural health centers (RHCs), and 140 rural health houses (RHHs) in its rural areas (Noorbala et al., 2017).

### 2.2 Study setting

The research team carried out their study in Semnan and Garmsar cities as the two most populated cities of the province because there was some evidence about the weaknesses in providing mental health services in urban areas compared to rural areas, where most of the province's population reside (Dadfar & Bahrami, 2015; Sharifi, 2009). Due to the hierarchical and pyramid PHC system in Iran, which defines UHCs as common system referral component (third level), the list of eligible UHCs was obtained from Semnan University of Medical Sciences. These UHCs included centers active in the provision of mental health services as well as those with a permanently on‐site general practitioner (GP) and mental health expert (psychiatrist). Then, based on the mental health service providers’ level of cooperation and willingness to participate in the study, 12 UHCs were selected from Semnan and Garmsar. All the study activities, such as recruitment, pretest and post‐test, and intervention, were carried out in these select UHCs.

### 2.3 Study design

A two‐armed, single‐blind, randomized, controlled trial was conducted to test a TCBT developed for presentations of common mental health problems among general population referred to health centers in one of the Iran's province. TCBI materials are using simple and concrete treatment methods to make sure local health providers with no mental health training background could learn and implement the techniques. Using this method increases the access of general population to proper treatments especially in low‐ and middle‐income countries.

TCBT was developed based on the review of the most common EBTs for mental health problems in low‐ and middle‐income countries offers different treatment and decisions using common forms of therapy for various mental health problems. This approach allows the provider to assess the primary mental problem, select specific elements and their order, specify element dose, and construct structural treatment according to the patients’ symptom or existing problems.

TCBT was developed to focus on three common mental health problems in Semnan Province, namely depression, obsessive compulsive disorder (OCD), and anxiety (Eftekhari et al., 2021). The existing analysis of elements of shared across EBT for these problem areas was reviewed by an EBT‐based team (made up of a psychotherapist, a clinical psychologist, and a psychiatrist) to develop a list of the most effective components for each problem (Chorpita et al., 2005; Clark & Tylor, 2009; Murray et al., 2014). For each mental health problem under study listed the simplest and most applicable (by intervention GHSP) and culturally acceptable therapies and prepared a training package considering the EBT/CETA elements (Murray et al., 2014).

The educational content was designed so that the intervention GHSP could acquire abilities to assess the participants, select the appropriate therapies/techniques, and implement them in at least eight weekly sessions lasting 45 to 60 min. All the intervention GHSPs were taught how to manage the therapy sessions, communicate properly, and apply the techniques they had learned. In addition to providing brief educational materials on psychopathology (cognitive, behavioral, and emotional) and CBT treatments available based on the DSM‐V, common CBT techniques and their worksheets were also taken into consideration (thought stopping, slow breathing, relaxation techniques, Socratic questioning, behavior activation, assigning homework, etc.).

A single‐blind design was used, and the interviewers were not informed about the group to which the participants belonged at the initial assessment. To perform the intervention, TCBT was provided by 14 non‐specialized general health service providers (GHSP) with at least a bachelor's degree in medical and health sciences (such as nursing, midwifery, and public health) who worked in the selected UHCs with no educational background in mental health. These 14 GHSPs received instructions and non‐psychiatric training on TCBT by licensed psychotherapists with good experience in cross‐cultural evidence‐based interventions (intervention GHSP). In parallel, 15 eligible trained GHSPs in the selected UHCs carried out the recruitment, baseline assessment, and post‐interviews assessment using the adapted study instrument; the follow‐up was also performed (assessment GHSP). The post‐assessment interviews were held after the treatment period was completed in both arms. The study was registered in the Iranin Registry of Clinical Trials, number IRCT20210609051527N1.

### 2.4 Participants

Study participants were recruited from individuals attending UHCs during December 2018 to September 2019 and residing in Semnan and Garmsar cities. As part of the health screening process, the mental health of all adult individuals who attend UHCs in Iran was routinely measured by utilizing the Kessler Psychological Distress Scale (K6) (Hajebi et al., 2018). Based on the study protocol, those attendances who were tested positive by applying the K6 (K6 score ≥10) were precisely informed about the nature and purpose of the study and invited to participate. The inclusion criteria were: (1) being at least 18 years old and (2) depression or anxiety or OCD scores higher than 14, 17, and 6 vise versa based on GHSP screening tool results (Eftekhari et al., 2021). The screening results of the GHSP tool was considered as the baseline assessment of the study participants. The exclusion criteria consisted of active suicidal ideations, active psychosis or major developmental delay, currently receiving any services or treatments for mental health problems including medications.

### 2.5 Study instrument

Before beginning the intervention, a qualitative assessment was carried out to identify the common major mental health problems among people residing in Semnan and Garmsar. The free list technique was used on the general population, and interviews were held with key informants, which indicated how these people perceived, described, and prioritized mental health problems in their community (Eftekhari et al., 2021). According to the results of these interviews and their comparison with the mental health problems noted by people from the general population, depression, anxiety, and OCD were selected for developing an adapted mental health instrument for the trial study. A functional impairment checklist was also added to the instrument.

All the existing locally validated measures were reviewed by the research team based on the common mental health disorders selected. Three symptom‐based measures were selected to form the basis of the study instrument, as described below. Any prioritized symptoms/tasks or words/idioms derived from the qualitative study that were not already included in the selected measures were then added with minor adjustments.

For depression, the Persian version 21‐item Beck Depression Inventory was selected (Ghassemzadeh et al., 2005), and seven items were added to the modified version based on the results of the qualitative assessment. The added items covered religious beliefs, social networks, and digestive problems. In addition, the wording of some statements were updated, such as “I'm locked as a result of some serious thinking” (a Persian idiom meaning the inability to make decisions in the face of countless thoughts). The face, content, criterion, and construct validity (confirmatory factor analysis) of the designed tool were assessed to determine its validity (Eftekhari et al., 2019). Each item in this measure contained four statements; the respondents had to pick the one that best described their feelings over the past 2 weeks.

The Persian version 21‐item Beck Anxiety Inventory (Kaviani & Mousavi, 2008) was also modified for the assessment of anxiety. According to the qualitative assessment, the following six items were added to this inventory: aggression, sleep disorder, impatience/hurry, disturbing thoughts, lack of focus, and worry. A Likert‐type scale was used to indicate how much the respondents were bothered with each item over the past 2 weeks (“not at all” = 0 to “severely” = 3).

The Yale‐Brown Obsessive Compulsive Scale (Y‐BOCS) scale was used for assessing OCD (Esfahani et al., 2012). This modified 21‐item scale included obsessive thoughts and compulsive behavior, most of which were already mentioned by the participants in the qualitative assessment. The respondents expressed how much each specific thought/behavior bothered them (“never” = 0 to “always” = 3).

A locally developed functional impairment checklist was also included as a separate instrument. This checklist contained 25 different tasks that the participants in a previous qualitative study had said they regularly did to meet their own as well as their families’ needs. The respondents stated their current levels of difficulty of each task (“no difficulty” = 0 to “unable to do” = 4).

The validity and reliability of this instrument was tested based on the face, content (both qualitative and quantitative methods), and criterion validity methods. The impact score for all the items was between 1.8 and 5. CVR was between 0.7 and 1 for all the items, and S‐CVI/Ave was 97.09 ± 0.63. The mean relevance, clarity, and simplicity and the S‐CVI/Ave were 97.09 ± 0.63, 97.64 ± 0.61, 96.73 ± 0.7, and 98.2 ± 1.9, respectively. The final package was compared to the Structured Clinical Interview for DSM Disorders (SCID) for the criterion validity assessment. In this step, 160 patients who either had the set psychiatric diagnoses or were healthy and resided in the two selected cities entered the study (patients with depression: *n* = 40, anxiety: *n* = 40, OCD: *n* = 40 and healthy: *n* = 40). Cronbach's alpha coefficient was used for reliability, such as .92 for depression, .95 for anxiety, and .88 for OCD, suggesting the high reliability of all three dimensions. The cut‐off point was estimated based on the sensitivity and specificity analysis. For depression, an 89% sensitivity and 59% specificity were deemed suitable, which made for a cut point of 14. That is, if the sum of the scores of the items in the depression dimension exceeded 14, that person was considered positive in the screening test. Similarly, considering a sensitivity of 80% and specificity of 44% for anxiety, the cut‐off point was calculated as 17. Considering a sensitivity of 80% and sensitivity of 49%, a cut‐off point of 6 was also calculated for OCD.

For reliability assessment, Cronbach's alpha was calculated as .88 and the internal consistency coefficient as .7. For internal reliability assessment, Cronbach's alpha scores were more than .88 for all the items, which showed the high internal consistency among them (Dejman et al., 2021). A training workshop was performed for homogenization of the raters.

### 2.6 Intervention

#### 2.6.1 Modification of the intervention

This study was originally designed and approved as a joint project with Johns Hopkins University Bloomberg School of Public Health. The main objective of the study was to evaluate the CETA in Iranian adults and adapt it to their population. During the quantitative phase of adapting the study instrument in fall 2017, the sanctions imposed on Iran became a barrier against contacting Johns Hopkins University. For this reason—and the impossibility of using CETA‐based educational content and implementing the model in Iran—the research team decided to develop and adapt a transdiagnostic approach. Also, based on the CETA principles and elements (except for alcohol and drug abuse disorders), the research team considered the generally low income in the study setting. After an initial review—and in collaboration with mental health professionals from UMEA University in Sweden and the support, assistance and scientific advice of the US partners—the TCBT model was developed for training and implementation in the place of the CETA.

#### 2.6.2 Mental Health Services as Usual

All the controls were referred to receive Mental Health Services as Usual (MHSU) within the national PHC network. Since 1989, mental health services were adopted as one of the major components of PHC networks in Iran. In each UHC, GHSPs are responsible for screening the patients in terms of different elements of their health and provide appropriate and necessary healthcare based on standard instructions (Smith, 2020). Screening for all the elements of health and mental health takes place by valid and reliable instruments using interviews, the results of which are then sent to an online application called SIB.

The Kessler Psychological Distress Scale (K6) was used to assess mental health; in this application, the respondents rated their feelings over the past 30 days (“never” = 0 to “always” = 4). The cut‐off point was 10 and people who got a score above 10 were referred to a GP in the UHC for diagnosis and to be prescribed suitable medications (Ministry of Health and Medical Education, 2015). Considering the severity of their problems and type of mental health disorders, the GP generally referred the patients to the UHC's mental health provider (usually a psychologist) for three to four training and counseling sessions (approximately one session per month). These sessions consisted of simple and popular counseling techniques and general mental health advice within the scope of the nationally approved instructions, based on CBT. The mental health provider was also responsible for the follow‐up process and referral to GP whenever needed. In this study, the assessment GHSPs followed up on the process of MHSU treatment for the controls through monthly phone calls and completed the participant monitoring form for them.

#### 2.6.3 Intervention: TCBT

Transdiagnostic mental health interventions focuses on similar components across EBTs (e.g., cognitive processing) (Murray et al., 2014). Transdiagnostic approach required that these commonality treatment components be taught to health providers and provided them a guideline for which components to be used for which presenting problems (Murray et al., 2014).

All the intervention participants received TCBT—a transdiagnostic treatment approach inspired by CETA developed by two authors (DM and ASF) for delivery by counselors without any mental health education in a setting with few mental health professionals. For this study population, TCBT was designed to treat symptoms of common mental health problems including depression, OCD, and anxiety based on the priorities that came out during a prior qualitative study (Eftekhari et al., 2021). TCBT used in this study consisted of eight elements, which are listed and described in Table 1.

#### 2.6.4 Training workshops

Before the trial, the intervention GHSPs received TCBT training through a 6‐day workshop. Case vignettes and role‐playing were common methods applied by the trainers (the psychotherapists and the clinical psychologist) to allow the intervention GHSP ample opportunity for practice. After the workshop, each of the participants evaluated and treated two cases over 8 weeks under the supervision of the local clinical psychologist. The weekly supervision sessions were held in small groups during the pilot study and intervention phase. In each session, each case was discussed and reviewed separately to provide guidance if needed. All supervision sessions were held by the workshop trainers through social networks, and weekly notes were presented on the case reports.

After completing the pilot study, the intervention GHSPs participated in a 3‐day re‐training workshop held for reviewing the training materials, discussing the challenges and sharing experiences. All the participants in the intervention group received eight weekly TCBT therapy sessions.

##### Training materials

In the training materials, each component had a 1–3 page “guideline” and a 1‐page “actions sheet.” The actions sheets included both goals and example wording for many goals to provide extra guidance for the health providers. The action sheets were designed for the treatment sessions and were used and piloted during the training sessions. Core, cross‐cutting cognitive‐behavioral strategies were included in each component: (a) the “what” (e.g., element) and “why” (e.g., rationale); (b) in‐session, guided practice of elements (modeling; role‐plays); (c) daily homework assignment/review and problem‐solving completion barriers. All training materials were designed to be adjusted and used by the trained local health providers and supervisors’ feedback during and after training.

The selection and ordering of elements for problem areas involved integrating findings from existing EBT for adult anxiety, depression, and OCD treatment. EBTs for depression were predominantly focused on behavior activation. The elements used in TCBT vary depending on the client's symptom presentation and what the local trained counselors observed from the subjects. The health providers were taught strategies for identifying the main or primary problem area(s) for each subjects (e.g., depression and anxiety). This decision process was based on the subject's responses on locally validated assessment measures; clinical presentation (e.g., the symptoms presented by the subjects or that were observed from the subjects by the counselors); and (c) discussion with the supervisors who were expert in TCBT and had trained the health providers.

During training, brief case vignettes were presented, some with assessment results, to allow counselors to practice element selection and to implement the proper treatment method. Counselors had been worked in small groups to select the primary problem, and identify the TCBT elements for each case vignette. This activity allowed for building critical thinking around TCBT. Trainers worked in small work groups to observe progress, giving feedback, and understand the counselors’ thought processes.

Supervision groups between local counselors and local supervisors continued during the training sessions, pilot study, and intervention periods based on daily virtual meetings. The comprehensive model used included sharing the information of the subjects, element selection and techniques used, and feedback loops encouraging local counselors to modify the delivery of components to increase their skills and knowledge based on their ongoing experiences.

All cases were then reported on and discussed with TBCD trainers each week, who documented details of each case. In addition, the TCBT trainer and local supervisor discussed the counselors’ performance and jointly made decisions about the elements and technique used and needed additional treatments for individual subjects during weekly internet calls.

Consistency was ensured through a multitier review approach. Specifically, counselors tracked their fidelity by following their step sheets and checking each step on their step sheets. They also completed a monitoring form for each session that included documentation of the components delivered and the steps used for each element. During the virtual supervision groups, the supervisor reviewed the documents by checking the monitoring forms and objective reporting (e.g., “subjects had negative thoughts and worminess. I used relaxation exercises and positive thinking to reduce stress and make the subject aware of his thoughts”). This process allowed the supervisor to determine which technique within the component was selected and delivered correctly. Supervisors provided the reports of the sessions for each case, and the supervisor asked questions specific to the treatment process and how they were completed. If errors within a session occurred (e.g., failure to complete a step, step delivered incorrectly), the supervisor coached the counselor to redo this component or step during the following session.

### 2.7 Screening, assessments, and randomization

The enrollment of participants began in December 2018 and ended in September 2019, considering the intervention sample size. All the individuals who rated positive in the UHC's initial mental health screening using K6 were referred to an assessment GHSP to enter the study. As the assessment GHSPs were in charge of following up on all the participants (intervention and control groups), they were instructed about the study instruments, pretest and post‐test, inclusion and exclusion criteria, informed consent forms, randomization process, and monitoring of participants by follow‐up phone calls. They also completed the organized participant monitoring forms designed by the research team in a 2‐day training workshop.

After the introduction process and providing information about the study and its objectives, the assessment GHSPs conducted the screening interviews using the study instrument. All the participants who obtained a score at least above the cut‐off point for the examined mental health problems were enrolled as the trial group. To participate in the trial, the subjects had to sign consent forms; if they were illiterate, the assessment GHSPs read the consent form to them and obtained their signature. The participants entered the randomization, and their screening results were recorded as the baseline measurements.

The assessment GHSPs randomly assigned an ID number to each participant in the treatment (TCBt) or control (MHSU) groups. A third independent researcher made the random ID number list using computer‐generated random numbers. This list included a sequence of C (Control) and T (Treatment) using balanced block randomization with four blocks. The details of the block series were unknown to all the researchers and GHSPs. The random allocation to the intervention or control groups was also blinded (unknown to assessment GHPS prior to participating). As the information about randomization was inserted on the allocation form, which was kept only by the independent researcher, all the GHSPs and their supervisors remained blinded to the randomized assignment; selection bias was thus reduced.

In this trial, the assessment GHSPs followed up on the participants by phone calls (weekly in the intervention group and monthly in the control group) to assess the treatment process, cases of withdrawal and contact information. The post‐test interviews were conducted after the completion of the treatment (8 weeks in the intervention group and 3 to 4 months in the control groups) by the assessment GHSPs.

### 2.8 Sample size

To calculate the sample size, a pair‐wise comparison was carried out and the intervention (TCBT) and control (MHSU) conditions were compared on the primary outcomes. The effect size analyses were used to calculate the sample size and test power. To achieve a moderate effect size (.50), each arm would need 100 participants. Considering test power = .80, alpha = .05, a drop‐out rate of 20%, and a 1:1 ratio of intervention to control group members, 300 participants were included in this RCT (*n* = 150 per group). Given the rates of symptomology in the target population, the estimates showed that approximately 500 people needed to be screened in order to identify 300 eligible and willing participants.

### 2.9 Statistical analysis

Univariate descriptive statistics were used to describe the samples. Bivariate analyses, including the *t*‐tests and chi‐square tests, were applied to examine the association between the potential continuous and categorical confounding variables, respectively, and the group assignment. The paired‐sample *t*‐test was used to compare the mean of the mental health variables at the end of the intervention compared to before. This procedure was applied within each group. The changes in each variable were also calculated as the difference between the values at the end of the treatment and baseline. The mean changes were compared between the treatment and control groups using general linear models. All the analyses were performed in STATA version 15.1 (StataCorp, College Station, TX).

### 2.10 Ethical considerations

The study protocol was approved by the Research Ethics Committee of the National Institute for Medical Research Development (NIMAD) under the code IR.NIMAD.REC.1395.047 and the Research Committee of the University of Social Welfare and Rehabilitation Sciences (IR.USWR.REC.1395.113). All participants signed the consent form or verbally gave their consent to participate in the study. They all fully understood the nature of the trial and their own role and contribution, were ensured about the voluntary nature of their involvement and about their ability to withdraw from the trial at any stage without any repercussions.

## 3 RESULTS

A total of 520 patients with positive Kessler Psychological Distress Scale (K6) were willing to participate in this project. All of the candidates signed inform consent forms and completed the study instruments at baseline. A total of 61 people had scores below the cut‐off point and 459 people (231 in the MHSU group and 228 in the TCBT group) ultimately entered the study and were randomized.

The mean age of the participants was 34.93 years (SD = 10.95). A total of 82% of the participants (*n* = 374) were married and 87.8% (*n* = 403) were female. As for education, 45.3%, 29.2%, and 25.5% of the participants had primary, intermediate, and high school education or above, respectively. All the data on the participants, including assessment scores, date of enrollment and follow‐up notes, were recorded in the monitoring forms provided by the assessment GHSPs. Table 1 shows the baseline characteristics of the study population by group. The results revealed no significant differences between the intervention and control groups in terms of the potential confounders (age, gender, marital status, and education; *p *> .05, Table 2).

**TABLE 1:** TCBT elements

Component	Brief description	Inclusion
Engagement (encouraging participation)	Attention to perceived/logistical barriers to engagement	Provided to all participants
Psych education (introduction)	Normalization of symptoms problems Program information (duration, content, expectations)	Provided to all participants
Anxiety management (relaxation)	Strategies to reduce physiological tension/stress	Included as optional if client presented with physiological symptoms of anxiety or OCD
Behavioral activation (getting active)	Identifying and engaging in pleasurable, mood‐boosting activities	Included as optional if subject presented with symptoms related to depression
Cognitive coping/restructuring	Identifying and connecting thoughts, feelings, and behaviors Evaluating and restructuring thoughts to be more accurate and/or helpful	Provided to all participants
In vivo exposure, live exposure	Innocuous: Facing and confronting systematically and gradually intrusive/obsessive thoughts Gradual desensitization	Included in many EBT for symptoms related to obsessive thoughts and for all EBT for anxiety and OCD disorders Included as optional
Imaginal gradual exposure	Facing obsessive thoughts and associated thoughts and feelings Gradual desensitization/exposure	Aspects of imaginal exposure and thoughts included in all EBT for symptoms related to OCD problems (variation across EBT in method) Included in all cases at these sites due to OCD
Safety (suicide/homicide/danger assessment)	Assessing risk for suicide, homicide, and etc	Provided to all participants, used as needed

*Abbreviations*: EBT, evidence‐based therapy: OCD, obsessive compulsive disorder.

**TABLE 2:** Description of the baseline characteristics of the study population by group

	MHSU (control)	TCBT (intervention)	
	*N* = 231	*N* = 228	*p*
Age, mean ± SD	34.54 (10.9)	35.3 (11.01)	.45
Gender, *n* (%)			.53
Female	205 (88.7)	198 (86.8)	
Male	26 (11.3)	30 (13.2)	
Marital Status, *n* (%)			.85
Married	189 (81.8)	185 (81.1)	
Single	42 (18.2)	43 (18.9)	
Education, *n* (%)			.32
Primary	112 (48.5)	96 (42.1)	
Intermediate	61 (26.4)	73 (32)	
Higher education	58 (25.1)	59 (25.9)	

*Abbreviations*: MHSU, Mental Health Services as Usual; OCD, obsessive compulsive disorder; TCBT, transdiagnostic cognitive behavioral therapy.

### 3.1 Subject withdrawal

At any point during the study, all the subjects were free to withdraw without any repercussions in terms of their future care. Documentation about whether or not each subject completed the clinical study had been recorded by the assessment GHSPs.

**FIGURE 1:**
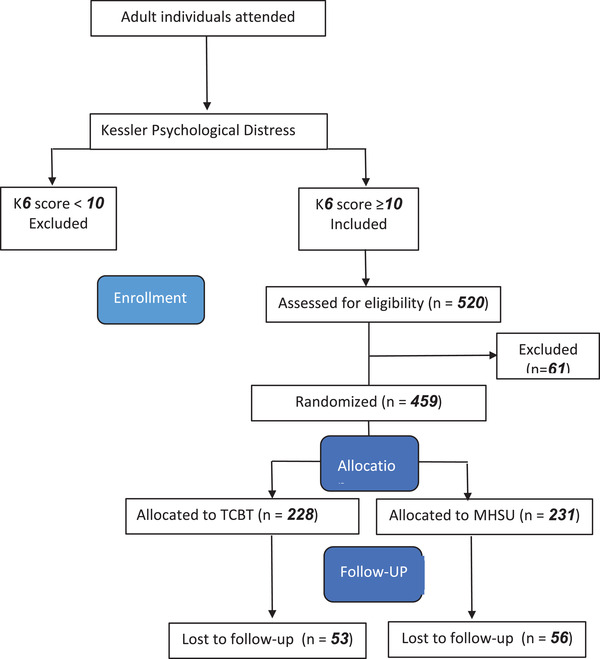
Flow diagram of participants throughout the study

A total of 109 subjects, 53 persons in the TCBT group and 56 in the MHSU group, withdrew from the treatment (Figure 1). The withdrawn subjects were followed up 1 week later by the assessment GHSPs to examine whether their symptoms had worsened or if any adverse events had happened.

There was no significant difference between the intervention and control groups in terms of the potential confounders after withdrawal. The intervention GHSPs discontinued cooperation with the participants at any time if their clinical judgment showed a medical emergency. In this study, only one patient exited the study for this reason. Immigration from the neighborhood (22 subjects), unwillingness to continue the treatment due to family problems such as relatives’ illness (5 subjects), husbands’ non‐consent (7 subjects), and referral to a private psychiatric based on their desire (5 subjects) were the reasons for their withdrawal from the study. Sixty‐nine subjects did not respond despite constant follow‐up by the assessment GHSPs. The majority of withdrawn subjects happened after the middle of treatment period (in both groups).

The timing of post‐test assessment was at the end of the treatment, both in the control and intervention groups. All the participants were re‐assessed using the study instruments after 8 weeks in the TCBT group and after 4 months in the MHSU group by the assessment GHSPs. Table 3 shows the mean mental health scores before and after the intervention in each group.

**TABLE 3:** Descriptive statistics, mean (SD), and net effect (95% CI) of mental health scores, pre‐, and post‐intervention by TCBT and MHSU groups

Mental health problems	MHSU (*N* = 175)	TCBT (*N* = 175)	Net effect (95% CI)
Depression_(Pre)	18.83 (7.30)	18.79 (7.14)	
Depression_(Post)	8.08 (7.81)	5.89 (5.91)	
Pre‐post change	−10.75 (9.45)***	−12.90 (7.87)***	−2.15 (0.55–3.74)***
Anxiety_(Pre)	27.95 (11.56)	27.61 (11.58)	
Anxiety_(Post)	11.94 (11.60)	8.00 (7.86)	
Pre‐post change	−16.02 (13.62)***	−19.60 (12.07)***	−3.58 (1.22−5.94)***
OCD_(Pre)	10.12 (5.16)	10.22 (5.13)	
OCD_(Post)	4.35 (4.42)	3.36 (3.86)	
Pre‐post change	−5.77 (5.77)*	−6.86 (5.55)*	−1.09 (0.04−2.11)*
Function_(Pre)	22.16 (18.19)	21.92 (17.41)	
Function_(Post)	11.96 (15.02)	8.89 (13.40)	
Pre‐post change	−10.19 (16.13)*	−13.03 (14.66)*	−2.84 (0.007−5.66)*

*Note*: *p < .05, ***p < .001, pre and post intervention comparison for mental health variables within each group.

*Abbreviations*: MHSU, Mental Health Services as Usual; OCD, obsessive compulsive disorder; TCBT, transdiagnostic cognitive behavioral therapy.

Comparison of Means (SD) of Depression in pre‐ and post‐treatment situation within each group (TCBT−MHSU) showed that the treatment was effective in both groups (diff Depression in MHSU [C] and TCBT [T]: −10.75 and −12.9), and also that there was statistically significant difference between TCBT and MHSU groups in the mean difference (*p *< .001).

Comparison of Means (SD) of anxiety in the pre‐ and post‐treatment situation within each group (TCBT−MHSU) showed that the treatment was effective in both groups (diff Anxiety in TCBT and MHSU: −16.02 and −19.6), and also that there was statistically significant difference between TCBT and MHSU group in the mean difference (*p* < .001).

Comparison of Means (SD) of OCD in the pre‐ and post‐treatment situation within each group (TCBT−MHSU) showed that the treatment was effective in both groups (diff OCD in TCBT and MHSU: −5.77 and −6.86); however, the mean difference among intervention group was statistically significantly higher than among the MHSU group (*p* < .05).

Comparison of Means (SD) of function in the pre‐ and post‐treatment situation within each group (TCBT−MHSU) showed that the treatment was effective in both groups (diff function in TCBT and MHSU: −10.19 and −13.03). Also, the mean difference in the intervention group was statistically higher than in the MHSU group (*p *< .05).

## 4 DISCUSSION

The increasing number of studies regarding CBTs suggests that this type of treatment is transferable, adaptable, acceptable, and effective (Bolton et al., 2014; Murray et al., 2014; Weiss et al., 2015). The present study employed the TCBT method inspired by CETA. The reason for the change in the study approach from CETA to TCBT was the impossibility of the presence of the CETA team in Iran due to the sanctions. Similar to CETA, this approach has been proposed for use by all GHSPs in the PHC system. The transdiagnostic approach is a powerful approach for making changes in the treatment process. This study design allowed us to make appropriate decisions about the effects of TCBT as well as its elements, execution mechanisms, and planning for implementation in similar contexts. The results of this study indicate that this approach can be practiced in all health centers of Iran for three reasons:

*First*: TCBT is a flexible method and is used as a suitable approach for controlling and treating comorbidities with a single approach (Stancliffe et al., 2008). This method teaches us how, when, and to what extent to treat comorbidities. This method equips GHSPs with skills that enables them to well manage and treat the patient's symptoms and reactions.

*Second*: TCBT saves time and reduces the resources needed for training GHSPs; this approach is thus very cost‐effective and reduces the gap in the provision of necessary treatments for mental health problems by preventing the provision of repetitive training and expanding the necessary training based on problems.

*Third*: TCBT can reduce the impact of cultural differences in the use of standard diagnostic classifications such as DSM‐V and ICD‐11 (Canino & Alegría, 2008), because identifying problems in its context is necessary for designing and implementing appropriate interventions, and TCBT is well capable of these steps. For instance, during a qualitative study, one may encounter mental health problems that are not well differentiated according to the DSM‐V and ICD‐11 gold standards; that is, the choice of treatment based on the conventional methods does not enable a proper response and timely treatment; therefore, the use of TCBT will be quite helpful in these cases.

This study aimed to evaluate the effectiveness of the TCBT approach in reducing common mental health problems in comparison with MHSU in adults in the Semnan Province of Iran. Based on the results, the symptoms of common mental health disorders, including depression, anxiety, and OCD, were reduced significantly with the application of the TCBT approach compared to the conventional methods. Nevertheless, interpreting these results must be pursued with extreme caution because, in this study, the proposed intervention was compared with the conventional treatments only as a clinical trial. Moreover, despite the adequate sample size in the study, the possibility of generalizing the results is limited due to the use of convenience sampling. Besides, data collection and provision of weekly interventions by the GHSPs can falsely create social acceptance, and this frequent communication can lead to data bias. Also, treatment follow‐up by a screener (assessment GHSPs) based in the center adds to this bias. In other studies where this follow‐up was performed by out‐of‐center screening, this bias was less pronounced (Bolton et al., 2014; Murray et al., 2014).

In expressing the strengths of the study, it should be noted that the presence of the GHSPs in the heart of the community and their close relationship with people lead to mutual trust in the participants; therefore, information was easily collected from 228 participants in the intervention phase of this study in the two cities of Semnan and Garmsar. Meanwhile, in some studies, GHSPs were not located in the health center and were located in other areas, such as the hospital, which limited the patients’ visits due to the associated stigma. Furthermore, the implementation of the study and the process of the interventions were closely monitored in this study by visiting the health center every week and also via online technologies on other days of the week. Nonetheless, the long distance from the observer's domicile to the site of the interventions caused some limitations. For instance, time and transportation and accommodation costs have been among the weaknesses of the monitoring process. Similar challenges were faced in the study conducted on the same subject in Iraq (Aarons & Sawitzky, 2006).

### 4.1 Strengths

Although the intervention site in this study was restricted to Semnan and Garmsar cities, which limited participants’ access, the challenges of this process were greatly reduced due to the very favorable cooperation of the healthcare network management team.

### 4.2 Limitations

Regarding the number of missing cases, despite the continuous and weekly follow‐up of the screeners (assessment GHSPs), the number of withdrawals was almost equal to 24% of the total study population, which, due to its proportional distribution in the control and intervention groups, did not affect the study findings. Regarding the duration of follow‐up in this study, due to the lack of longitudinal follow‐up and its limitation to the last session of the intervention, the possibility of judging the long‐term effects and reliability of the interventions and the generalizability of the results will be limited. Therefore, it is recommended that this limitation to be considered in similar studies and that the necessary measures be taken to enable longer follow‐ups.

## 5 CONCLUSION

The transdiagnostic approach has been proposed for service delivery by GHSPs in the PHC system. The researchers hope that, by implementing this approach, the limitations of the existing treatments and the challenges of education, treatment, and supervision in the field of mental health can be minimized. The positive results of these studies give GHSPs the motivation to change their treatment approach and acquire the necessary skills. Obviously, due to the lack of specialized personnel (psychologists and psychiatrists) in many remote areas, the implementation of this approach can be considered an effective step in promoting communities’ mental health and reducing their related problems.

## AUTHOR CONTRIBUTIONS

Katayoun Falahat, Monir Baradarn Eftekhari, Masoumeh Dejman, Zohreh Mahmoodi, and Ameneh Setareh Forouzan contributed to the design of the study. Masoumeh Dejman and Ameneh Setareh Forouzan supervised the study. Monir Baradarn Eftekhari, Katayoun Falahat, Masoumeh Dejman, and Mojgan Padyab contributed to the acquisition, analysis, and interpretation of data. Katayoun Falahat, Monir Baradarn Eftekhari, Masoumeh Dejman, and Zohreh Mahmoodi contributed to drafting of this manuscript and revising. Samira Tavassoli contributed to translating and revising manuscript final draft. All authors approved the final version of the manuscript.

## CONFLICT OF INTEREST

The authors declare no conflict of interest.

### PEER REVIEW

The peer review history for this article is available at https://publons.com/publon/10.1002/brb3.2551

## Data Availability

The data that support the findings of this study are available from the corresponding author upon reasonable request.
